# The Impact of Virtual Reality (VR) Gaming and Casual/Social Gaming on the Quality of Life, Depression, and Dialysis Tolerance in Patients With Chronic Kidney Disease: A Narrative Review

**DOI:** 10.7759/cureus.44904

**Published:** 2023-09-08

**Authors:** Danyel Gurz, Kavya Coimbatore Dada, Veeramachaneni Naga Nyshita, Fiyinfoluwa D Aderibigbe, Mankaranvir Singh, Kameshwar P Yadav, Sanjay K Shah, Binali Pumbhadia, Kiran Abbas, Wajiha Khan, Vaishnavi Kumaran

**Affiliations:** 1 Department of Internal Medicine, Combined Military Hospital Lahore, Lahore, PAK; 2 Department of Internal Medicine, Davao Medical School Foundation, Davao City, PHL; 3 Department of Internal Medicine, Andhra Medical College, Visakhapatnam, IND; 4 Department of Pediatrics, All Saints University Dominica, Roseau, DMA; 5 Department of Medicine and Surgery, Government Medical College, Patiala, IND; 6 Department of Internal Medicine, Universal College of Medical Sciences, Bhairahawa, NPL; 7 Department of Internal Medicine, Janaki Medical College, Janakpurdham, NPL; 8 Department of Internal Medicine, Government Medical College, Surat, IND; 9 Department of Community Health Sciences, Aga Khan University, Karachi, PAK; 10 Department of Oncology, Ziauddin University, Karachi, PAK; 11 Department of Public Health, McMaster University, Ontario, CAN

**Keywords:** quality-of-life, dialysis tolerance, general nephrology dialysis and transplanation, digital game therapy, exergaming, virtual reality simulation, chronic kidney disease (ckd)

## Abstract

This comprehensive narrative review aims to investigate the impact of virtual reality (VR) and social gaming on the quality of life, depression, and dialysis tolerance in patients with chronic kidney disease (CKD), a growing global health concern affecting the quality of life and increasing mortality rates. Through a combination of exercise and video games, exergaming, and digital game therapy (DGT), sedentary risks can be mitigated and therapy adherence enhanced. Despite potential side effects such as exhaustion and nausea, research indicates that virtual therapeutic games improve motivation and dialysis tolerance, and even reduce brain activity in pain-associated regions during procedures. These methods are superior to traditional distraction techniques like music, movies, and toys. Exergaming, social gaming, and VR gaming can improve various health factors including depressive symptoms, functional capacity, muscle strength, fatigue, physical activity, mental health, motivation, engagement, cognition, and overall quality of life for CKD patients. Representing a novel approach to CKD management, these interventions promote physical activity, cognitive stimulation, and social interaction. Continued research and innovation will facilitate the integration of VR-based therapies into routine CKD care.

## Introduction and background

Chronic kidney disease (CKD) is a global health issue that is becoming more prevalent due to factors such as the rapidly aging population and high incidence of diabetes and high blood pressure [1-2]. CKD leads to muscle wasting and decreased endurance, known as uremic sarcopenia [3]. This condition affects the quality of life and increases mortality rates [4]. Physical exercise has been recommended to improve various health indicators in CKD patients, including arterial stiffness, nutritional markers, inflammation, and kidney function [4]. Combining aerobic activity with strength training is effective in treating CKD [5]. However, adherence to exercise programs can be challenging. In light of this, virtual reality (VR) gaming is being explored as an alternative technique to enhance motivation and adherence [6].

Electronic gaming has traditionally been played with a gamepad, joystick, keyboard, or mouse, where there is only minimal physical movement. With the use of computer-based VR systems, a person can respond to stimuli in the environment and interact with them in real time. These systems can be quite sophisticated, providing the player with a view of the world across a 360-degree viewing area, or very straightforward simulation of a virtual environment on a flat-screen television using a commercial gaming system (such as the Nintendo Wii or Xbox Kinect). Virtual gaming platforms also give players the chance to interact in settings or carry out tasks that would otherwise be inaccessible or of personal significance [6]. Exergaming, a term for playing video games while concurrently exercising, is a combination of the words “exercise" and "gaming." These activities instill gradual encouragement in patients to become more active, improve one's reaction time, attention span, level of alertness, memory, and overall cognitive abilities, minimize the negative effects on the environment and social obstacles, lessen depression symptoms, bring about a high level of satisfaction, and foster a sense of familial connection. Exergames hold the potential to enhance health results from both a mental and physical standpoint [7-8].

A comprehensive review of energy usage in exergaming has reported that it boosts heart rate, oxygen consumption, and energy use when at rest, while also encouraging light-to-moderate physical activities [9]. A range of healthcare professionals are utilizing VR technology to improve patient treatments and offer numerous novel interventions such as Xbox 360 and Nintendo Wii that call for patient attention and active engagement in multiple sclerosis, rheumatoid arthritis, diabetes, spinal cord injury, and stroke patients [8,10]. This comprehensive narrative review was conducted to examine the impact of VR gaming and casual/social gaming on the quality of life, depression, and dialysis tolerance in patients with CKD.

## Review

The emergence of virtual reality and exergaming

A rising amount of research supports the idea that excessive sedentary behavior is bad for human health. Despite the benefits of quick access to information and communication, prolonged screen time has been linked to health and psychological issues in young children, adolescents, and infants [11]. The World Health Organization (WHO) has characterized excessive digital gaming as a condition when unfavorable symptoms start to manifest. According to research, spending an inordinate amount of time in front of screens increases the risk of obesity, high blood pressure, and malfunction of high-density lipoproteins, which are also significant risk factors for cardiovascular morbidity [12-14].

Exergaming is a portmanteau word that combines gaming with fitness. Exergame is a term with a multitude of interpretations, indicative of the various methods employed by diverse research groups across the last two decades. Terminologies such as physical gaming, exertainment, active video games, and active video training have been introduced by health researchers to describe the motivation for physical activity or involvement during video game play. Exergaming, as described by Bogost as "the combination of exercise and video games," is defined as "the process of gaming in any digital device" although the term "exercise" is open to several interpretations [15,16].

Without necessarily referring to the extent of body movement involved, exercise can refer to the process of improving one's ability to perform a set of actions. Exergaming in this context might be defined as the deliberate, controlled practice of honing reaction times in a purely sedentary environment. The eye-hand coordination skills of professional players in multiplayer online combat arena games are improved, practiced, and honed [17]. Therefore, it can be inaccurate to use the term "exercise" in the same sentence as "physical activity" or "physical fitness" while discussing exergaming systems. According to Oh and Yang, exergaming is defined as "playing exergames or any video games that require physical exertion or movements that are more than sedentary activities and also include strength, balance, and flexibility activities." Immersive VR-based exercise games attempt to close the gap between unplanned, unstructured, and repetitive body movements and the components of a training session [15].

Improvements in muscle strength and force can be achieved through various types of muscle contractions [18]. The most frequent contractions seen in daily life include static isometric contractions, and eccentric and concentric contractions that together enable dynamic movements (like lifting and lowering an object). VR-based motion tracking systems can promote the execution of isometric contractions by motivating users to perform a flawless plank, a well-known exercise recognized for its ability to fortify core muscles and improve stability [18].

VR is a new technology that immerses users in a realistic, lifelike environment that has been artificially constructed. End users use a helmet-mounted display (HMD), which utilizes tracking technology and physical motion, such as eye or head motions, to move around a digitally created virtual environment. This virtual world may reproduce complex science-fiction vistas or genuine environments [19]. It has been demonstrated that using distraction techniques like music, movies, and toys can help people feel less pain and anxiety. The most recent form of providing diversion during medical procedures, VR is more effective than other traditional techniques (cognitive behavior therapy and music therapy). VR typically consists of a computer-generated environment, and three-dimensional use and orientation are possible. Children were the target audience for VR since they are more drawn to imaginative play and activities. VR is typically classified into non-immersive, completely immersive, semi-immersive, and augmented VR, all of which are frequently utilized in therapeutic settings to divert patients and promote patient relaxation [20-22].

In therapeutic and healthy populations, VR training (VRT) applications using gaming consoles like the Nintendo Wii, Sony PlayStation EyeToy, Microsoft Kinect, and Dance Revolution are regarded as appealing, inspiring, and encouraging exercise concepts [23]. This supplementary and alternate training method could fill the gap between exercising and playing video games, or "exergaming." Exergaming, which makes use of a VR setting, has been used for therapeutic (such as cardiac rehabilitation, neuro-rehabilitation, and musculoskeletal system disorders) and general improvement of physical fitness. The resulting energy expenditure of exercise games typically ranges from light to moderate depending on the nature of the underlying bodily movements [24-27].

Virtual reality gaming in healthcare

Patients on hemodialysis (HD) typically experience symptoms including weariness, nausea, dizziness, and headaches, which usually appear during their dialysis treatments. Without having any negative effects, the Joviality VR program reduced symptom severity. VR applications provide a secure platform to enhance the dialysis patient experience [28]. The feasibility, acceptability, and effectiveness of a novel exergame program to increase daily physical activity in diabetes patients on HD therapy are all enhanced by a non-immersive VR exercise program [29]. Elderly people receiving dialysis have reported fewer depression symptoms because of VR gaming [5]. VR has been utilized in medicine to help patients regain their motor and cognitive abilities. It is successful in enhancing physical abilities including posture, balance, and motor skills [30].

Physical exercise in combination with VR improved hemodialysis patients' functional abilities and quality of life [31]. For CKD patients who experience discomfort during hemodialysis, VR gaming can provide a distraction, reducing their perception of pain, fatigue, nausea, lightheadedness, and headaches and easing workout continuity. Regarding overall adherence, the study's participants attended approximately 74.4% (19.1 sessions) of all the sessions made available to them. Within the VR-control group (VRC), attendance was notably higher, with approximately 82.8% (16.2 sessions) of the offered sessions attended. On the other hand, the control-VR group's (CVR) attendance rate was slightly lower at approximately 63.2% (17.0 sessions) of the sessions provided. These findings indicate distinct variations in adherence levels between the VRC and CVR groups in the study [28]. The intervention group (IG) exhibited a 53% increase in posture transitions from sitting to walking and a 39% increase in transitions from standing to walking compared to the control group (CG). Additionally, the IG showed significantly reduced sedentary behavior compared to the CG. In summary, the IG spent 5% less time in sitting and lying positions and 47% more time in standing and walking activities than the CG. The key innovation of VR pertains to its practicality during the HD process, addressing limitations seen in prior exercise interventions for HD patients [32].

The potential for a cognitive stimulation intervention during hemodialysis is discussed in a literature review. It emphasizes how crucial cognitive development is for these people to carry out everyday tasks and make decisions. Significant gains in processing speed, sustained attention, alertness, visuospatial working memory, cognitive flexibility, immediate and delayed visual memory, and visual motor spatial coordination were reported in a review of the research on digital gaming for seniors [29].

The main benefit of cognitive stimulation intervention over nurse-supervised intradialytic exercise is the lack of a need for ongoing care from a physical therapist or skilled nursing personnel. Implementing an exercise intervention during conventional hemodialysis therapy may become easier [28]. Digital game therapy (DGT) can be seen as advantageous, giving older patients receiving hemodialysis a cutting-edge instrument with growing potential in the field of mental health. Through social interaction and encouragement during their exercise sessions, CKD patients can engage with others through VR gaming [29].

The impact of virtual reality gaming on quality of life and depression

VR has demonstrated efficacy in reducing depression symptoms and improving the quality of life in individuals with CKD, due to its versatile applications in patient care. Specifically, it has been shown to improve functional capacity, muscle strength, fatigue, physical activity, depression, and quality of life across a wide age range [28-33].

In comparison to nurse-supervised intradialytic exercise treatment, the data suggests that virtually overlooked intradialytic exercise activity is just as effective in reducing depressive symptoms [28,32,33]. One study indicated that both the virtual and standard exercise groups showed a significant reduction in depressive symptoms following a four-week program, but the optimal intervention dosage and longevity of effects remain unknown. Integrating such intradialytic exercises into regular dialysis clinic procedures may notably lower the risk of depression [28].

Physical exercise, when combined with VR, demonstrated the ability to enhance functional capacity and various domains of quality of life in patients undergoing hemodialysis, especially those related to the physical domains [32]. An eight-week virtual reality exercise program (VREP) yielded significant improvements in leg strength, back strength, flexibility, and balance. Furthermore, it was shown that fatigue significantly decreased in the VREP exercise group, likely due to improved physical fitness, reducing perceived fatigue [34]. VR can also effectively distract patients undergoing HD from uncomfortable chairside medical procedures, reducing the intensity of symptoms like nausea and lightheadedness by diverting focus away from typical side effects, and it may also reduce brain activity in regions associated with pain responses [19]. According to another study, a six-minute 360-degree nature movie viewed using VR headsets can considerably enhance mental health and lower heart rate [35]. However, once the exercise program is discontinued, the achieved improvements are no longer maintained [36].

Virtual reality gaming and dialysis tolerance in CKD patients

The need for dialysis was expected to double by 2030, with 2.62 million people receiving it globally in 2010 [37]. Patients undergoing hemodialysis must go to dialysis clinics three times a week, spending four hours per session [28]. When surveyed, monotony was reportedly the primary complaint among hemodialysis patients [32], highlighting a need for new strategies to boost motivation and treatment compliance [38].

Hemodialysis is where the uncomfortable arterio-venous fistula technique is most frequently utilized [19]. Immersive VR is an emerging method that potentially lessens the discomfort that patients typically experience during hemodialysis sessions. Wearing a VR headset before undergoing a venous fistula puncture significantly decreased subjective pain scores and pain anxiety. Even if the pain level increased for the two sessions from its initial level, employing VR significantly lessened suffering as compared to receiving conventional nursing care. But another important indicator of how effectively a VR experience manages pain is the level of immersion during a session. This outcome may be explained by the idea that VR diverts attention away from unpleasant stimuli by changing how the environment is perceived. VR might lessen brain activity in areas where pain responses have been observed [19]. The usage of VR headsets to watch six-minute nature videos indoors led to noticeably lower sympathetic nerve activity and heart rate along with enhanced positive emotions, which improved psychological responses in dialysis patients [35].

However, VR can cause exhaustion, nausea, dizziness, and general ill-feeling, which are signs of cybersickness [19]. The symptoms of cybersickness resemble those in hemodialysis patients, such as fatigue, nausea, dizziness, and headaches brought on by intradialytic hypotension, osmotic changes, or other reasons. As a result, VR may make nausea and other common side effects of hemodialysis worse. However, a study found that before and after the first exposure to VR, there were significant reductions in symptoms like fatigue, nausea, oculomotor symptoms, disorientation, and overall symptomatology. However, during the second exposure, no statistically significant variations in scores were seen [19]. The precise mechanism as to how chairside VR exposure could minimize side effects from hemodialysis is unknown. Thanks to its high-definition graphics and interactive virtual objects, VR has the potential to be a great tool for distracting patients and shifting stimulus away from typical side effects, lessening the severity of symptoms like nausea and lightheadedness. Therefore, VR programs might be a secure option to reduce the discomfort in dialysis patients [19].

The intradialytic exergaming (IE), which is not nurse-supervised, automatically directs the patient through the exercise procedure, requiring hemodialysis patients to rotate their ankle joints within a specified range and at a specific pace to finish the exercise activity, and wearable and interactive interface technologies provide real-time visual and aural feedback through which patients can assess their strengths and weaknesses. Customizable exercise activities based on the patient's motor and intellectual capacities are possible with the IE, thereby minimizing the need for ongoing nursing care, lowering the workload, and reducing the usage of specific medical resources and associated micro costs [28,32].

IE enhances patient compliance and physical activity during hemodialysis thanks to its interactive, engaging nature. It is well-tolerated, safe, and motivates patients, thereby improving health outcomes and quality of life, especially for chronic kidney failure (CKF) patients. Significant benefits, such as improved physical fitness, body composition, and reduced fatigue, manifest after eight weeks of thrice-weekly sessions of IE, although these gains are lost if exercise ceases. IE notably improves the Timed Up and Go (TUG) performance by 18% and is well-tolerated throughout dialysis sessions. Notably, IE significantly reduces post-dialysis potassium rebound in the last 30 minutes, warranting further research on the optimal timing for intradialytic exercise [28,32].

Social gaming and its impact on CKD patients

Social gaming and DGT, such as Nintendo Wii-based exergaming, can enhance the quality of life for pediatric and elderly CKD patients undergoing hemodialysis [5]. These interventions improve functional capacity, muscle strength, physical activity, mental health, and cognitive abilities, and reduce depressive symptoms and fatigue, thereby enhancing patients' quality of life. As digital technologies advance further, the potential of DGT as a complementary tool in rehabilitation settings becomes more evident, relieving distress, anxiety, sadness, and isolation. Therefore, there is a need to further assess DGT's impact on depressive symptoms and cognitive disorders, thereby offering new resources for CKD management [29].

## Conclusions

There is significant evidence highlighting the remarkable benefits of VR and exergaming in managing CKD, improving the patient experience during hemodialysis by fostering enjoyment, social interaction, motivation, exercise adherence, and dialysis tolerance while relieving symptoms. This innovative approach encourages physical activity, cognitive stimulation, and social engagement, ushering in a new era in CKD management. Further research is needed to fully understand VR and social gaming's potential in CKD management, addressing the knowledge gap between modern and previous treatment guidelines. As VR technology continues to evolve, it could provide personalized, interactive workouts aiding CKD patients in maintaining active lifestyles, improving muscle strength, and combating muscle wasting. Anticipated advancements in VR technology aim for affordability, portability, and user-friendliness, which would increase accessibility for CKD patients in various settings. This access, combined with ongoing research and innovation, will accelerate the integration of VR therapies into standard CKD care.
